# Stimulation of Eryptosis and Hemolysis by Adrenic Acid Involves Oxidative Stress, Calcium Elevation, and Metabolic Collapse

**DOI:** 10.3390/ijms27104327

**Published:** 2026-05-13

**Authors:** Feryal H. Alharthy, Jawaher Alsughayyir, Mohammad A. Alfhili

**Affiliations:** Department of Clinical Laboratory Sciences, College of Applied Medical Sciences, King Saud University, Riyadh 12372, Saudi Arabia

**Keywords:** adrenic acid, PUFA, eryptosis, anemia, CVD

## Abstract

Omega-6 polyunsaturated fats (ω-6 PUFAs) are vital for many physiological functions, but their impact on cardiovascular disease (CVD) risk is controversial. Eryptosis alters blood viscosity by providing a procoagulant surface and leads to anemia, which is a recognized risk factor for CVD. This study examines the toxic mechanisms of adrenic acid (ADR), an ω-6 PUFA enriched in inflammatory and oxidative conditions, in red blood cells (RBCs). Purified RBCs were prepared from healthy volunteers and treated with 10–100 μM of ADR for 24 h at 37 °C under various physiological conditions. Eryptotic markers were studied through flow cytometry including Ca^2+^ (Fluo4/AM), loss of volume (forward scatter), phosphatidylserine (PS) exposure (annexin-V-FITC), and oxidative stress (H_2_DCFDA). Moreover, hemolytic markers were measured by colorimetric methods, whereas cellular morphology was visualized using a scanning electron microscope. ADR led to significant Ca^2+^ elevation, cell shrinkage and schistocyte formation, PS externalization, hemolysis, and oxidative stress. While guanosine, heparin, and NSC 23766 prevented eryptosis and hemolysis, melatonin, ATP, adenine, and L-NAME only prevented eryptosis. Conversely, mannitol and urea exacerbated eryptosis, whereas caffeine, mannitol, and urea under Ca^2+^ deprivation and membrane potential dissipation aggravated hemolysis. ADR induces erythrocyte membrane injury and eryptosis through Ca^2+^ elevation, oxidative stress, and metabolic exhaustion subject to inhibition by the Rac1 GTPase/NOS/COX pathway. Altogether, these findings present a novel mechanistic link between lipid dysregulation and RBC dysfunction which may improve dietary strategies to prevent and manage CVD.

## 1. Introduction

Adrenic acid (ADR; C22:4, ω-6), also named 7,10,13,16-docosatetraenoic acid, is an ω-6 polyunsaturated fatty acid (PUFA) abundant in human tissues including the adrenal gland, kidney, brain, and vascular system [1]. ADR is synthesized via the elongation of arachidonic acid (20:4, ω-6) or the elongation and desaturation of linoleic acid (18:2, ω-6) and can be converted back into arachidonic acid through β-oxidation [2]. Despite evidence suggesting a protective function for ω-6 PUFAs in cardiovascular disease (CVD), which is a primary contributor to global disease burden [3], outcomes from clinical trials and observational studies remain incongruent [4]. Therefore, it is highly recommended to avoid indiscriminately grouping all ω-6 PUFAs together and instead to examine the health impacts of each species in isolation due to their distinct biological functions. In this context, conflicting correlations between individual C-6 PUFAs and mortality were identified. For instance, the shorter ω-6 PUFA, linolenic acid, is linked to a diminished risk of cardiovascular mortality, unlike ADR [5].

A positive correlation was observed between inflammatory markers and ADR in patients at high risk of coronary artery disease, with a higher mortality rate compared to healthy controls [5]. Another study found that individuals with elevated serum ADR are at an increased risk of aortic stroke, myocardial infarction, and coronary heart disease [2]. Brouwers et al. [6] reported that ω-6 PUFAs should not be classified as purely proinflammatory, as they can have potent anti-inflammatory effects as well. Importantly, a previously unappreciated anti-inflammatory and pro-resolving effect of ADR was reported, offering an emerging therapeutic candidate in inflammatory conditions such as atherosclerosis, autoimmune diseases, asthma, and cancer.

Alongside the established common CVD risk factors, such as hypertension, obesity, hyperlipidemia, diabetes, smoking, and alcohol consumption, emerging studies indicate that anemia correlates with an elevated risk of CVD [3]. Anemia, resulting from a reduced lifespan of erythrocytes, is intimately linked to CVD [7] and increases the risk of developing CVD [8]. Recently, much focus has been directed toward the role of red blood cells (RBCs) in CVD, including heart failure, hypertension, atherosclerosis, and metabolic syndrome [9,10]. In particular, eryptosis plays a significant role in the onset of anemia, inflammation, and thrombosis across many clinical scenarios including CVD [11,12]. Eryptosis is primarily characterized by phosphatidylserine (PS) exposure, cellular shrinkage, and elevated intracellular Ca^2+^ concentrations. Moreover, the oxidative metabolites of ADR can elevate Ca^2+^ levels, a signal permissive of hemolysis and eryptosis. Nonetheless, there is a paucity of evidence regarding the effect of ADR on RBCs. Therefore, this study aims to examine the cytotoxic effects of ADR and to elucidate the associated molecular pathways.

## 2. Results

### 2.1. ADR Stimulates Eryptotic and Hemolytic Injury in RBCs

**Figure 1:**
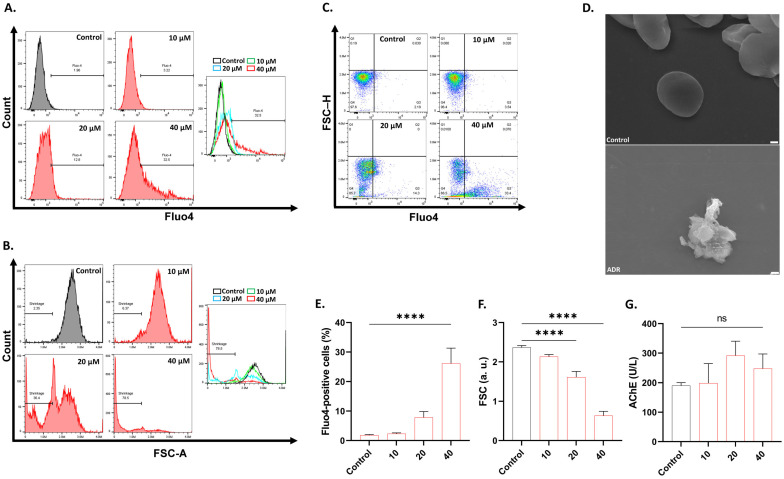
ADR elevates Ca^2+^ levels. (**A**) Fluo4 histograms. (**B**) FSC histograms. (**C**) Association of Fluo4 with FSC. (**D**) Schistocyte and membrane blebbing morphology (×5000. Scale bar: 1 μm) following treatment with 0 and 40 μM of ADR. (**E**) Percentage of cells with excess Ca^2+^. (**F**) FSC in arbitrary units (a.u.). (**G**) AChE activity. Cells were treated with 10–40 μM of ADR for 24 h at 37 °C. Results are shown as means ± SD (*n* = 9). **** (*p* < 0.0001).

The loss of cellular volume after intracellular Ca^2+^ elevation is an integral mechanism of erythrocyte death. Fluo4 fluorescence, FSC histograms, and dot plots of FSC-H and Fluo4 are shown in Figure 1A, Figure 1B and Figure 1C, respectively. Additionally, the morphological examination of cells after treatment with 40 μM of ADR showed red cell fragments (schistocytes) as well as membrane blebs, as illustrated in Figure 1D. In ADR-treated cells, the percentage of Fluo4-positive cells was significantly increased (*p* < 0.0001; Figure 1E) while FSC was significantly reduced (*p* < 0.0001; Figure 1F). No effect on AChE activity was observed (Figure 1G).

**Figure 2:**
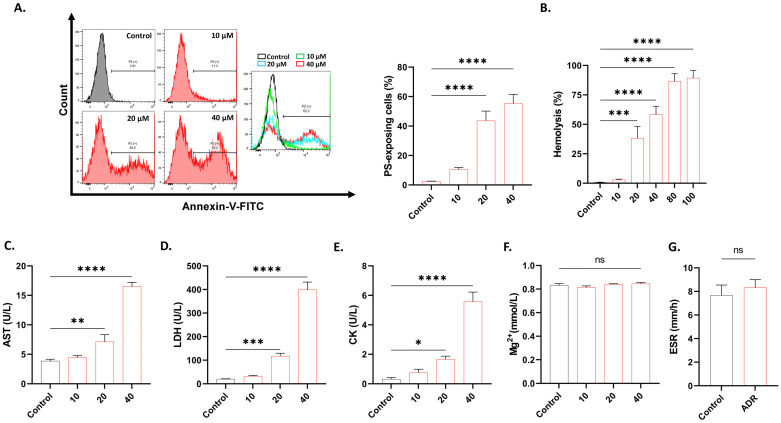
ADR elicits eryptosis and hemolysis. (**A**) Annexin-V-FITC histograms and percentage of eryptotic cells. (**B**) Percent hemolysis. (**C**) AST activity. (**D**) LDH activity. (**E**) CK activity. (**F**) Mg^2+^ levels. (**G**) ESR (control and 40 μM). Cells were treated with 10–40 μM of ADR for 24 h at 37 °C. Results are shown as means ± SD (*n* = 9). * (*p* < 0.05), ** (*p* < 0.01), *** (*p* < 0.001), and **** (*p* < 0.0001).

Ca^2+^ nucleation and shrinkage are markers of eryptosis, and PS translocation was next assessed using the annexin-V-FITC binding assay. Figure 2A shows that PS-exposing cells are significantly increased in treated cells (*p* < 0.0001). Furthermore, Ca^2+^ entry may also trigger membrane rupture. Indeed, we found that ADR treatment significantly increased the proportion of hemolytic cells (*p* < 0.0001; Figure 2B). Consequently, significant elevations (*p* < 0.0001) in the enzymatic activities of AST (Figure 2C), LDH (Figure 2D), and apparent CK-coupled enzymatic leakage signals (Figure 2E) were noted. However, Mg^2+^ levels (Figure 2F) and ESR (Figure 2G) were unchanged upon ADR exposure.

### 2.2. Oxidative Stress Contributes to ADR-Induced RBC Toxicity

**Figure 3:**
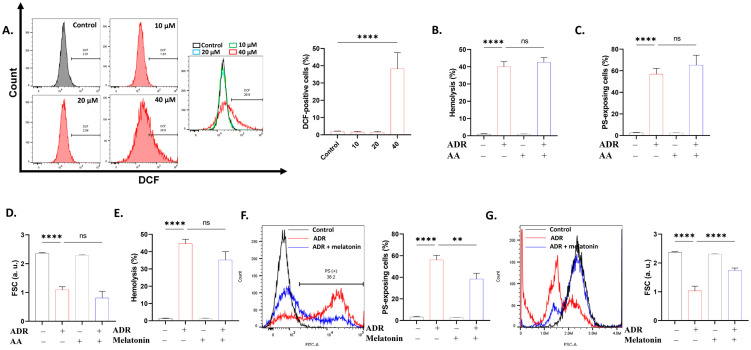
ADR stimulates oxidative stress. (**A**) DCF histograms and percentage of cells with oxidative stress. Effect of ascorbic acid (AA; 1 mM) on (**B**) hemolysis, (**C**) PS translocation, (**D**) and FSC. Effect of melatonin (5 μM) on (**E**) hemolysis, (**F**) PS translocation, and (**G**) FSC in cells exposed to 40 μM of ADR. Cells were treated with 10–40 μM of ADR for 24 h at 37 °C. Results are shown as means ± SD (*n* = 9). ** (*p* < 0.01) and **** (*p* < 0.0001).

Oxidative injury may precede eryptosis and hemolysis. Our results show that DCF-positive cells were significantly increased (*p* < 0.0001) following ADR treatment (Figure 3A) indicating increased oxidant burden. Next, to delineate whether antioxidants ameliorate ADR toxicity, cells were co-treated with vitamin C and melatonin. While vitamin C had no appreciable effect (Figure 3B–D), melatonin, while not reversing hemolysis (Figure 3E), significantly decreased PS translocation and cell shrinkage (Figure 3G).

### 2.3. Differential Regulation of ADR Toxicity by Osmotic and Ionic Manipulations

**Figure 4:**
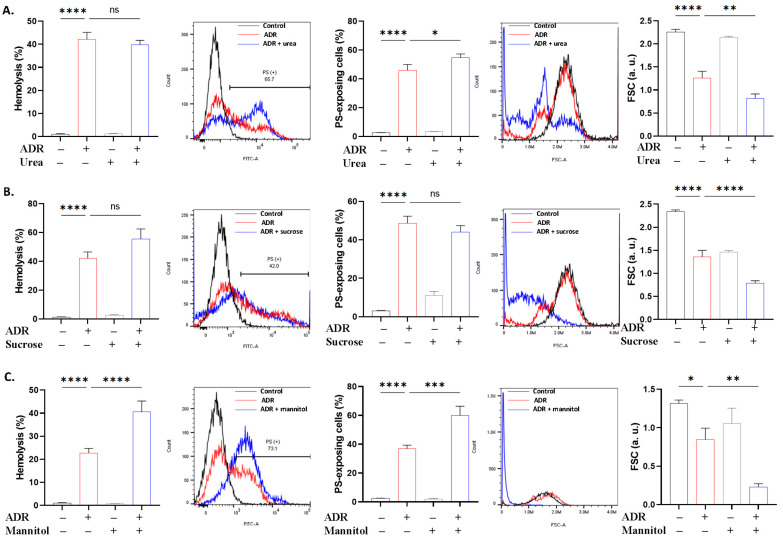
ADR toxicity under hyperosmotic stress. Effect of (**A**) urea (300 mM), (**B**) sucrose (250 mM), and (**C**) mannitol (284 mM) on hemolysis, PS translocation, and FSC in cells exposed to 40 μM for 24 h at 37 °C. Results are shown as means ± SD (*n* = 9). * (*p* < 0.05), ** (*p* < 0.01), *** (*p* < 0.001), and **** (*p* < 0.0001).

It was next of interest to explore whether ADR relies on a colloid-osmotic mechanism using permeant and impermeant osmolytes. Urea (Figure 4A) significantly aggravated ADR-induced PS exposure (*p* < 0.05) and cell shrinkage (*p* < 0.01) but not hemolysis. Whereas sucrose (Figure 4B) only exacerbated cell shrinkage (*p* < 0.0001), mannitol (Figure 4C) rather augmented all toxic endpoints including hemolysis (*p* < 0.0001), PS translocation (*p* < 0.001), and cell shrinkage (*p* < 0.01).

**Figure 5:**
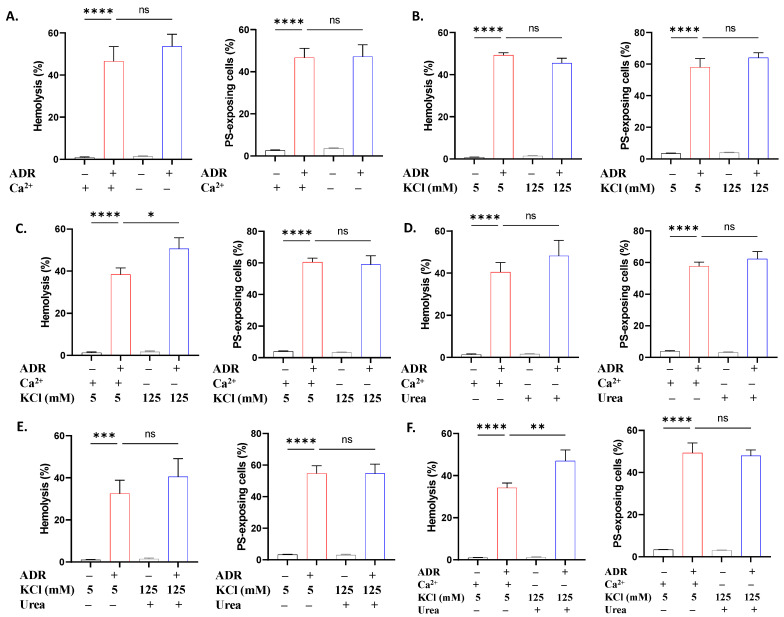
ADR toxicity following Ca^2+^ deprivation and K^+^ gradient dissipation. Effect of (**A**) Ca^2+^ deprivation, (**B**) KCl (125 mM), (**C**) Ca^2+^ deprivation and KCl (125 mM), (**D**) Ca^2+^ deprivation and urea (300 mM), (**E**) KCl and urea, and (**F**) Ca^2+^ deprivation, KCl, and urea on hemolysis and PS translocation in cells exposed to 40 μM of ADR for 24 h at 37 °C. Results are shown as means ± SD (*n* = 9). * (*p* < 0.05), ** (*p* < 0.01), *** (*p* < 0.001), and **** (*p* < 0.0001).

Based on the involvement of Ca^2+^ and loss of volume in ADR action, we probed their requirement by modifying the extracellular environment. Removal of extracellular Ca^2+^ (Figure 5A) and the increase in KCl to 125 mM (Figure 5B) did not significantly impact the cytotoxicity of ADR. Surprisingly, a dissipating K^+^ gradient in the absence of Ca^2+^ significantly increased the hemolytic potential of ADR (*p* < 0.05; Figure 5C). Furthermore, the addition of urea either in the absence of Ca^2+^ (Figure 5D) or together with K^+^ enrichment (Figure 5E) had no effect. Curiously, when Ca^2+^ was removed and both 125 mM KCl and urea were added, the hemolytic activity of ADR significantly increased (*p* < 0.01; Figure 5F).

### 2.4. Metabolic Substrates Selectively Modulate ADR-Induced RBC Death

**Figure 6:**
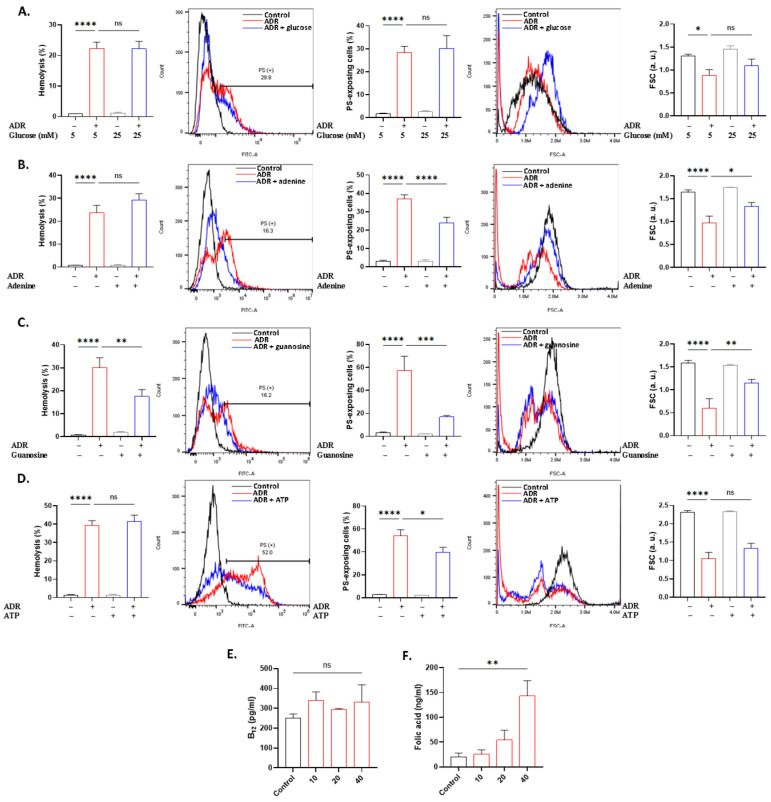
ADR toxicity following energy replenishment. Effect of (**A**) glucose (50 mM), (**B**) adenine (2 mM), (**C**) guanosine (2 mM), and (**D**) ATP (0.5 mM) on hemolysis, PS translocation, and FSC in cells exposed to 40 μM of ADR for 24 h at 37 °C. Intracellular levels of (**E**) B_12_ and (**F**) folic acid following treatment with 10–40 μM of ADR for 24 h at 37 °C. Results are shown as means ± SD (*n* = 9). * (*p* < 0.05), ** (*p* < 0.01), *** (*p* < 0.001), and **** (*p* < 0.0001).

Energy depletion primes cells for death. To investigate whether ADR action involves metabolic shutdown, we co-treated the cells with ADR in the presence and absence of energy substrates. The results show that the addition of 50 mM glucose instead of 5 mM offered no protection (Figure 6A), while adenine reversed both PS translocation (*p* < 0.0001) and shrinkage (*p* < 0.05), as seen in Figure 6B. Guanosine exhibited the strongest inhibitory effect on ADR toxicity as it significantly ameliorated all three toxic endpoints (Figure 6C), whereas ATP (Figure 6D) only blunted PS translocation (*p* < 0.05). Moreover, B_12_ utilization (Figure 6E) did not seem to be influenced, whereas intracellular folic acid (Figure 6F) was significantly increased (*p* < 0.01).

### 2.5. Heparin and Caffeine Exert Divergent Effects on ADR Cytotoxicity

**Figure 7:**
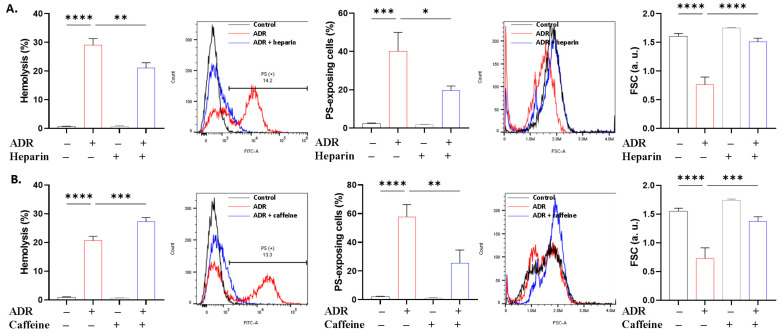
Protective effect of heparin and caffeine. Effect of (**A**) heparin (0.2 g/L) and (**B**) caffeine (0.5 mM) on hemolysis, PS translocation, and FSC in cells exposed to 40 μM of ADR for 24 h at 37 °C. Results are shown as means ± SD (*n* = 9). * (*p* < 0.05), ** (*p* < 0.01), *** (*p* < 0.001), and **** (*p* < 0.0001).

Heparin and caffeine have been reported to exert cytoprotective effects [13,14] through ill-defined mechanisms. Figure 7A demonstrates that heparin, like guanosine, conferred protection against all three toxic endpoints. Caffeine (Figure 7B), on the other hand, aggravated hemolysis (*p* < 0.001) but rather alleviated PS translocation (*p* < 0.01) and cell shrinkage (*p* < 0.001).

### 2.6. Inhibition of Key Enzymes Attenuates ADR-Induced RBC Injury

**Figure 8:**
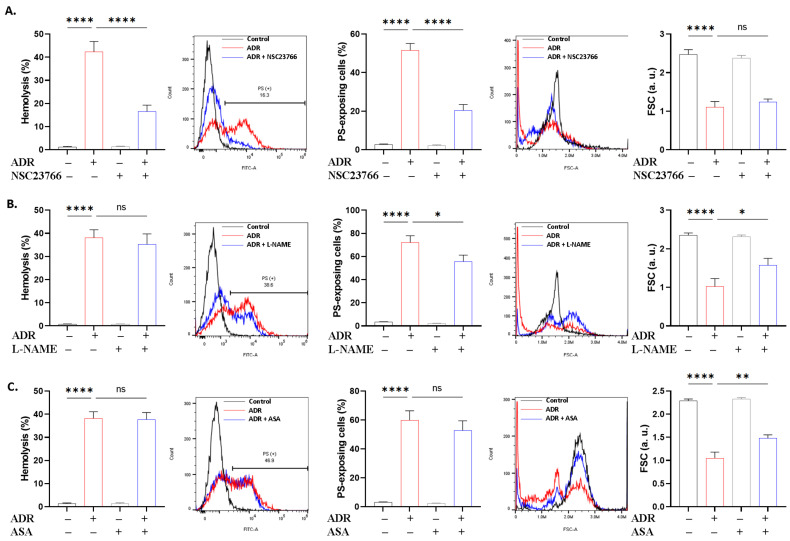
Involvement of Rac1, NOS, and COX in ADR toxicity. Effect of (**A**) NSC23766 (100 μM), (**B**) L-NAME (20 μM), and (**C**) acetylsalicylic acid (ASA; 25 μM) on hemolysis, PS translocation, and FSC in cells exposed to 40 μM of ADR for 24 h at 37 °C. Results are shown as means ± SD (*n* = 9). * (*p* < 0.05), ** (*p* < 0.01), and **** (*p* < 0.0001).

**Figure 9:**
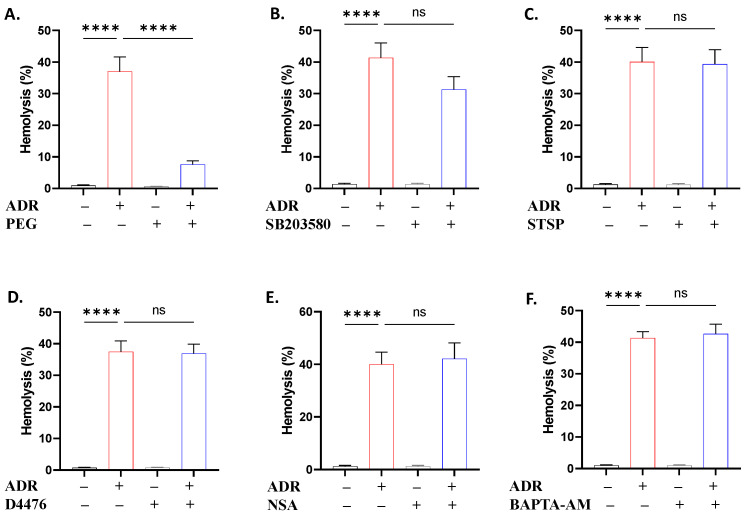
ADR hemolytic activity is abrogated by PEG. Percent hemolysis with and without (**A**) polyethylene glycol 8000 (PEG; 10 g/dL), (**B**) SB203580 (100 μM), (**C**) staurosporine (STSP; 1 μM), (**D**) D4476 (20 μM), (**E**) necrosulfonamide (NSA; 0.5 μM), and (**F**) BAPTA-AM (20 μM) in cells exposed to 40 μM of ADR for 24 h at 37 °C. Results are shown as means ± SD (*n* = 9). **** (*p* < 0.0001).

**Figure 10:**
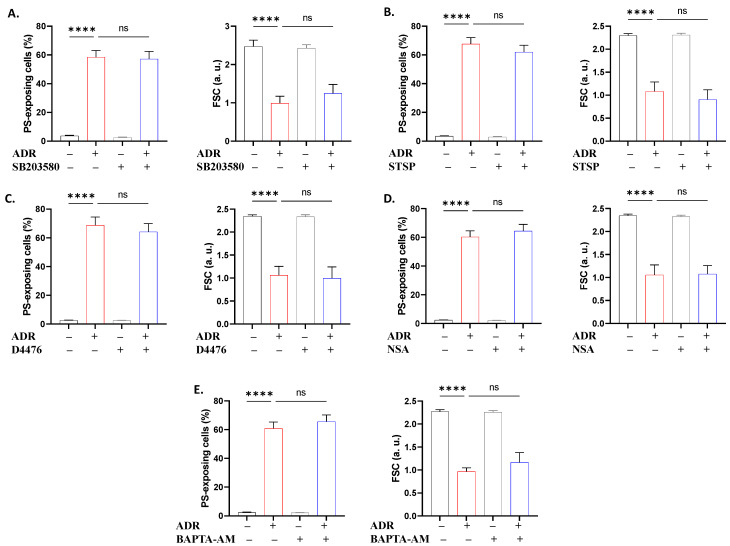
Inhibitors with no effect on eryptosis. Percent of eryptotic cells and cell shrinkage with and without (**A**) SB203580 (100 μM), (**B**) staurosporine (STSP; 1 μM), (**C**) D4476 (20 μM), (**D**) necrosulfonamide (NSA; 0.5 μM), and (**E**) BAPTA-AM (20 μM) in cells exposed to 40 μM of ADR for 24 h at 37 °C. Results are shown as means ± SD (*n* = 9). **** (*p* < 0.0001).

To define the molecular pathways governing ADR action, we performed a set of experiments using small molecule inhibitors. It was revealed that NSC23766 (Figure 8A) prevented hemolysis and PS translocation (*p* < 0.0001), L-NAME (Figure 8B) was effective against PS translocation and shrinkage (*p* < 0.05), and ASA (Figure 8C) only reversed the loss of volume (*p* < 0.01). Except for the anti-hemolytic activity of PEG (Figure 9A), none of the remaining inhibitors used offered significant protection against hemolysis (Figure 9B–F) or PS translocation (Figure 10).

## 3. Discussion

The role of the erythrocyte in CVD and metabolic syndrome is increasingly being recognized as mounting evidence implicates accelerated eryptosis as a significant contributor to anemia and microvascular complications in various clinical conditions. Since elevated ADR levels are associated with several risk factors for CVD, dissecting the isolated cytotoxic effects of ADR aids in mapping novel lipid-mediated pathological RBC clearance.

This study reveals a previously unrecognized cellular activity of ADR, which is the stimulation of hemolysis and eryptosis. ADR exposure increases cytoplasmic Ca^2+^ levels, which triggers a cascade of events that end in eryptotic loss of volume (Figure 1), hemolysis, and PS translocation (Figure 2). In particular, cell shrinkage is the result of the Gardos effect, which is the loss of KCl and water through the Gardos channel (KCNN4) in response to Ca^2+^ activation. Alternative mechanisms that similarly activate the Gardos K^+^ channel include oxidative stress (Figure 3), mechanical injury (Figure 1 and Figure 2), and metabolic collapse (Figure 6). This is clinically relevant, as cellular dehydration is pathognomonic of sickle cell disease for which, in addition to ischemic stroke, the Gardos channel blocker, senicapoc, has proven to be effective [15].

Although ADR exposure resulted in increased Ca^2+^ levels (Figure 1), chelation with BAPTA-AM failed to ameliorate hemolysis (Figure 9) and eryptosis (Figure 10). This observation suggests that Ca^2+^ elevation is involved, as opposed to required, in ADR-induced erythrocyte injury. As such, Ca^2+^ appears to represent a secondary mechanism underlying membrane integrity and phospholipid perturbation rather than a primary causal driver like oxidative stress (Figure 3) or metabolic exhaustion (Figure 6) as demonstrated by functional assays. Interestingly, in previous studies, Ca^2+^ deprivation attenuated eryptosis without appreciable increases in Ca^2+^ levels [16,17], which may reflect transient Ca^2+^ mobilization. Taken together, the current data do not support Ca^2+^ channel blockers as effective adjuncts to minimize ADR toxicity in erythrocytes.

The ultrastructural inspection of ADR-treated cells showed schistocytes (Figure 1), which is indicative, though not exclusive, of microangiopathic hemolytic anemia [18] that could lead to the enhanced adhesion of RBCs to the endothelium [19], increasing the risk of CVD through microthrombi. Indeed, schistocytes are seen in various RBC disorders and represent a morphological indicator of systemic microvascular damage [19,20]. Additionally, the appearance of membrane blebs represents a hallmark of eryptosis, due to the loss of cytoskeletal anchorage which creates weakened membrane regions that bulge outward as protrusions enlarged by phospholipid redistribution (Figure 2) [21]. Treated cells also demonstrate premature PS translocation (Figure 2), which has been reported to significantly increase the affinity of eryptotic cells to endothelial cells [22], adversely affecting rheological properties and causing microvascular injury, known risk factors of CVD.

Translocation of PS invariably leads to clearance of the dead cell from the circulation. The premature elimination of cells predisposes to anemia, as seen in renal disease and diabetes mellitus [23]. The RBC count may remain virtually constant if erythropoiesis is stimulated to match the rapid loss of eryptotic erythrocytes. When faster eryptosis cannot be entirely compensated by upregulated erythropoiesis, anemia occurs [24,25]. Accordingly, erythropoiesis-stimulating agents offer an opportunity to reduce the impact of ADR-induced eryptosis.

Heme, iron, hemoglobin, and other factors released from ruptured cells promote ROS production and inflammation, which is characteristic of atherosclerosis [26], further highlighting the detrimental effect of hemolysis induced by ADR. Although CK is not inherently found in RBCs, the leakage of adenylate kinase has been recognized as the source of interference in the CK assay, demonstrating membrane rupture [27]. Thus, the increase in activity observed herein is interpreted as an indicator of intracellular metabolite leakage through membrane lesions rather than true CK release.

Increased oxidative burden (Figure 3) is suggestive of overproduction of reactive oxygen species (ROS), which stimulates caspases [28] that degrade band 3, resulting in less interaction between band 3 and the cytoskeletal protein 4.2 in erythrocytes. This reduction facilitates the transfer of PS to the surface of the membrane [29,30]. Furthermore, PS translocation accelerates band 3 aggregation, leading to the subsequent phagocytosis of the eryptotic erythrocyte by macrophages [31]. Moreover, caspase-3 may influence eryptosis by regulating flippase activity [32]. Our findings align with those of Zhau et al. [33], who reported increased ROS generation in HepG2 cells exposed to ADR. Although vitamin C was ineffective against ADR toxicity, melatonin ameliorated PS translocation and restored cell volume (Figure 3). The antioxidant capacity of melatonin is facilitated by its function as a free radical scavenger [34] or an activator of antioxidant enzymes [35,36]. This indicates that ROS production may be indispensable to ADR toxicity in erythrocytes.

The observation that urea did not offer protection (Figure 4) seems to point toward the insensitivity of ADR to sphingomyelinase activity and ceramide production [37]. Instead, urea potentiated PS translocation and volume loss, suggesting the modification of the intracellular milieu to boost eryptosis. Moreover, mannitol increased ADR-induced hemolysis while sucrose did not. Since both are non-permeant osmolytes, it is therefore suggested that partial permeation of mannitol, whose size is half that of sucrose, occurred due to pore size selectivity, although additive or synergistic hyperosmotic stress cannot be excluded. On the other hand, the failure of sucrose to inhibit hemolysis implies that the hemolytic properties of ADR are not exclusively osmotic in nature. In contrast, the exacerbation of cell shrinkage is consistent with additive osmotic effects likely through Ca^2+^ elevation (Figure 1) and p38 MAPK [14,38].

When incubation media were modified by eliminating Ca^2+^, no effect was observed on ADR-induced hemolysis or eryptosis (Figure 5), indicating that Ca^2+^ influx is not necessary for ADR action. Similarly, a preserved K^+^ gradient is also not required, as evidenced by the unchanged toxicity in 5 and 125 mM extracellular KCl. However, upon introduction of both manipulations, with or without urea, hemolysis was potentiated, suggesting that lytic pathways triggered by ADR are modulated by extracellular ionic composition. In particular, under Ca^2+^ deprivation and elevated extracellular K^+^, activation of the Gardos channels is prevented and the transmembrane K^+^ gradient is dissipated, which abrogates volume regulatory capacity. In this environment, the membrane defects triggered by ADR may therefore render the cell more susceptible to osmotic lysis due to uncontrolled ion flux and water entry. Of note, caffeine (Figure 7) had a similar effect on hemolysis, and, in addition to its antioxidant properties, it has been shown to modulate Ca^2+^ channels [39]. Altogether, these data favor the formation of Ca^2+^-independent membrane lesions possibly through pore formation and colloid-osmotic lysis, as indicated by the reversal of hemolysis by PEG (Figure 9), similar to its effect on ω-3 FAs [40,41].

The divergent cellular pathways of hemolysis and eryptosis are highlighted by the contrasting effects of energy substrates on ADR toxicity. The inhibitory role of guanosine against both forms of cell death, as opposed to adenine and ATP being selectively effective against eryptosis (Figure 6), suggests that the lytic arm may have relied more on the antioxidant effect of guanosine [42], whereas metabolic shutdown was exclusively required by the eryptotic machinery. Notably, the increased accumulation of folic acid following ADR treatment seems to indicate perturbed cellular export or impaired utilization, potentially secondary to metabolic disruption (Figure 6). Since erythrocytes have limited metabolic capacity, this result suggests folate trapping and altered distribution as opposed to metabolic turnover. In this regard, heparin (Figure 7) mimicked the effect of guanosine, whereas caffeine only showed anti-eryptotic effects [43,44], lending support to the central role of oxidative stress in ADR action.

Signaling enzymes involved in the stress response of the erythrocyte to ADR include Rac1 GTPase, NOS, and COX (Figure 8) as suggested by functional assays using specific inhibitors. Since mature erythrocytes lack transcriptional activity, conventional genetic manipulation techniques are not feasible. Instead, pharmacological inhibition constitutes an approved functional approach to interrogate signaling pathways involved in cell death. Thus, the current results derived from inhibitor assays serve as indicators of enzymatic participation rather than definitive evidence of molecular targeting. Like other inhibitors, distinct ameliorative effects were revealed, with Rac1 being involved in the lytic and eryptotic arms of toxicity, while NOS mediated only eryptosis. Indeed, Rac1 is essential for preserving normal membrane deformability and mechanical stability [45] and plays a critical role in ROS production by activating NADPH oxidases [46], indicating a crosstalk between Rac1 and ROS (Figure 3) in ADR toxicity. NOS generates nitric oxide, which has opposing effects on eryptosis [47,48]. Moreover, the production of prostaglandin E_2_ is mainly mediated by the COX-2 pathway [49]. It has been previously demonstrated that prostaglandin E_2_ induces a reduction in cellular volume attributable to K^+^ efflux [50]. Taken together, our data suggest that Rac1, NOS, and COX interact with the Ca^2+^-K^+^ cascade and oxidative injury to bring about eryptotic and hemolytic effects. These results lend support to the divergent but partially overlapping cellular pathways mediating hemolysis and eryptosis. Rac1, being involved in both modalities, seems to occupy an upstream position and activate distinct downstream mediators depending on the type of cell death induced. In this case, the data support NOS in the eryptotic pathway but identified none in the hemolytic pathway. COX cannot be accurately attributed to either arm since cell shrinkage, like Ca^2+^ and ROS, can follow both types of cell death. Therefore, the ability of Rac1 inhibition to ameliorate hemolysis in contrast to NOS or COX inhibition suggests the participation of additional Rac1-dependent mechanisms other than these two downstream effectors, including metabolic exhaustion (Figure 6) and osmotic lysis (Figure 9).

## 4. Materials and Methods

### 4.1. RBC Culture

Experiments were performed according to the Eryptosis Study Consortium [13]. Heparin- and EDTA-anticoagulated blood samples were collected from twenty healthy subjects (twelve males and eight females) whose ages ranged from 24 to 38 years, and who had normal CBC and BMI results. All subjects provided written and verbal consent according to the Helsinki Declaration. RBCs were isolated by centrifugation (2500 RPM, 20 min), washed twice, and resuspended in PBS. Approval for the study was provided by the IRB of King Saud University (#E-23-7485). All compounds were supplied by Solarbio Life Sciences (Beijing, China). A stock solution of ADR (10 mM; 2.8 mg/mL DMSO) was prepared and stored in aliquots at –80 °C. Isolated RBCs (5% hematocrit) were subjected to ADR (10–100 μM) in Ringer solutions at 37 °C for 24 h. This range lies within the upper physiological to pathophysiological range previously reported for ADR in human plasma [51,52,53,54]. Distilled water was used as a positive control to induce complete hemolysis, whereas negative control cells were treated with the corresponding DMSO concentration used to solubilize the highest ADR concentration for each experiment (0.4–1.0%). This DMSO concentration is not toxic to RBCs, as seen from the results of control cells, which is consistent with previous observations [44,55].

### 4.2. Hemolysis

The supernatants were harvested by centrifugation (13,300× *g*, 1 min). The BS-240Pro analyzer (Mindray, Shenzhen, China) was used to assay extracellular Mg^2+^ and lactate dehydrogenase (LDH), aspartate aminotransferase (AST), and creatinine kinase (CK) activities. The LMPR-A14 microplate reader (Labtron, Surrey, UK) was used to measure hemoglobin at 405 nm [56] as follows:

$$\%Hemolysis=\;\frac{ADR-induced\;Hb\;release}{water-induced\;Hb\;release}\;\times 100$$

### 4.3. Intracellular Ca^2+^

Intracellular Ca^2+^ levels were quantified through flow cytometry (FCM) utilizing the Northern Lights flow cytometer (Cytek, Fremont, CA, USA). Washed cells were incubated with 2.5 μM Fluo4/AM in a 5 mM CaCl_2_ buffer at 37 °C for 30 min in the dark, and 10,000 events were recorded for Fluo4 intensity by stimulation at 488 nm and detection of emitted light at 520 nm [57].

### 4.4. Annexin-V-Binding and Forward Scatter

FCM was used to detect PS exposure. Briefly, washed RBCs stained with 1% annexin-V-FITC in 5 mM CaCl_2_ buffer were incubated for 10 min away from light, and 10,000 cells were analyzed for annexin-V-binding at 488 and 520 nm excitation and emission spectra, respectively. To exclude potential treatment-induced autofluorescence, unstained control and ADR-treated cells were examined, and no appreciable shift in FITC baseline fluorescence was observed. Forward scatter (FSC) readings were also obtained to estimate cell volume [58].

### 4.5. Oxidative Stress

Cells were stained with 10 μM 2′,7′-dichlorodihydrofluorescein diacetate (H_2_DCFDA) for 30 min at 37 °C in the dark to evaluate oxidative stress. DCF fluorescence intensity was measured at λ_max_ Ex/Em = 488/520 nm [59].

### 4.6. Erythrocyte Sedimentation Rate (ESR)

Samples were allowed to stand vertically in the Westergren tube for 1 h away from light as a marker of aggregation [60].

### 4.7. Cellular Morphology

Control and treated cells (40 µM ADR) were analyzed using an ultra-high-resolution scanning electron microscope (JSM-7610F, Jeol, Tokyo, Japan). Cells were fixed in 2.5% glutaraldehyde, stained with 1% osmium tetraoxide, dried in 50–100% ethanol, and examined [61].

### 4.8. Acetylcholine Esterase (AChE) Activity

The activity of AChE was measured in hemolysates using the BS-240Pro analyzer. In the reaction mixture, butyrylthiocholine undergoes hydrolysis by AChE to yield butyrate and thiocholine, which converts hexacyanoferrate (III) to hexacyanoferrate (II), resulting in a decrease in the absorbance at 405 nm [62].

### 4.9. Intracellular Vitamin B_12_ and Folate

Hemolysates were assayed for B_12_ and folate content using Mindray’s CL-1200i chemiluminescence analyzer.

### 4.10. Functional Assays

Hemolysis, PS translocation, and cellular volume were evaluated after treatment of cells with 40 μM ADR for 24 h. This concentration was used as it permits a robust response and mechanistic interrogation without confounding by excessive cell death. Inhibitors were maintained throughout this incubation window to assess their ability to modulate the final toxic endpoints under identical treatment conditions. Experiments were performed in the presence and absence of p38 inhibitor SB203580 (100 μM), PKC inhibitor staurosporine (STSP; 1 μM), CK1α inhibitor D4476 (20 μM), cell permeable Ca^2+^ chelator BAPTA-AM (20 μM), cyclooxygenase inhibitor acetylsalicylic acid (ASA; 25 μM), Rac1 GTPase inhibitor NSC23766 (100 μM), MLKL inhibitor necrosulfonamide (NSA; 0.5 μM), nitric oxide synthase (NOS) inhibitor L-NAME (20 μM), melatonin (5 μM), ascorbic acid (AA; 1 mM), ATP (0.5 mM), urea (300 mM), sucrose (250 mM), or mannitol (284 mM) [40].

### 4.11. Statistical Analysis

Results are shown as means ± SEM of three independent experiments, each conducted in triplicate. Statistical analyses were performed with Prism v9.5.1 (GraphPad, San Diego, CA, USA) using either Student’s *t*-test or one-way ANOVA. Statistical significance was established based on a *p* value of less than 0.05.

## 5. Conclusions

In conclusion, ADR is revealed as a novel stimulator of erythrocyte death through oxidative injury, Ca^2+^ elevation, energy depletion, and signaling nodes involving Rac1 GTPase, NOS, and COX (Table 1). Rac1 is the only enzyme that seems to mediate both hemolysis and eryptosis, whereas NOS inhibition selectively offered protection against eryptosis. COX, however, seems to reverse cell shrinkage without appreciable effect on hemolysis or PS exposure. An optimized approach targeting multiple molecular nodes is therefore likely to be required to effectively attenuate the erythrotoxicity of ADR. Altogether, this study offers new mechanistic insights linking anemia and microvascular dysfunction in CVD to accelerated RBC loss, and informs dietary and pharmacological interventions aimed at mitigating lipid-mediated erythrocyte injury in inflammatory conditions.

## Figures and Tables

**Table 1:** Modulators of adrenic acid-induced erythrocyte injury.

Inhibitor/Manipulation	Hemolysis	PS Translocation	Cell Volume
Guanosine	Decrease	Decrease	Increase
Heparin	Decrease	Decrease	Increase
Caffeine	Increase	Decrease	Increase
Mannitol	Increase	Increase	Increase
NSC23766	Decrease	Decrease	No effect
Melatonin	No effect	Decrease	Increase
L-NAME	No effect	Decrease	Increase
Adenine	No effect	Decrease	Increase
Urea	No effect	Increase	Decrease
Sucrose	No effect	No effect	Decrease
Acetylsalicylic acid	No effect	No effect	Increase
Ca^2+^ deprivation and dissipation of K^+^ gradient	Increase	No effect	No effect
Ca^2+^ deprivation, dissipation of K^+^ gradient, and urea	Increase	No effect	No effect
ATP	No effect	Decrease	No effect
PEG	Decrease	Not suitable	Not suitable
Vitamin C	No effect	No effect	No effect
Ca^2+^ deprivation	No effect	No effect	No effect
Dissipation of K^+^ gradient	No effect	No effect	No effect
Ca^2+^ deprivation and urea	No effect	No effect	No effect
Dissipation of K^+^ gradient and urea	No effect	No effect	No effect
SB203580	No effect	No effect	No effect
D4476	No effect	No effect	No effect
Staurosporine	No effect	No effect	No effect
BAPTA-AM	No effect	No effect	No effect
Necrosulfonamide	No effect	No effect	No effect
Glucose	No effect	No effect	No effect

## Data Availability

The data generated in the present study are available from the corresponding author, M.A.A., upon reasonable request.
